# Temperature-Modulated Micromechanical Thermal Analysis with Microstring Resonators Detects Multiple Coherent Features of Small Molecule Glass Transition

**DOI:** 10.3390/s20041019

**Published:** 2020-02-13

**Authors:** Maximilian Karl, Lasse H.E. Thamdrup, Jukka Rantanen, Anja Boisen, Thomas Rades

**Affiliations:** 1 Department of Pharmacy, University of Copenhagen, Universitetsparken 2, 2100 Copenhagen, Denmark; maximilian.karl@sund.ku.dk (M.K.); jukka.rantanen@sund.ku.dk (J.R.); aboi@dtu.dk (A.B.); 2 Department of Health Technology, Technical University of Denmark, Ørsteds Plads, 2800 Kgs. Lyngby, Denmark; lhth@dtu.dk; 3 Danish National Research Foundation and Villum Fondens Center for Intelligent Drug delivery and sensing Using microcontainers and Nanomechanics (IDUN), Ørsteds Plads, 2800 Kgs. Lyngby, Denmark

**Keywords:** thermal analysis, modulated, MEMS, resonator, string, glass transition, mode shape, indomethacin

## Abstract

Micromechanical Thermal Analysis utilizes microstring resonators to analyze a minimum amount of sample to obtain both the thermal and mechanical responses of the sample during a heating ramp. We introduce a modulated setup by superimposing a sinusoidal heating on the linear heating and implementing a post-measurement data deconvolution process. This setup is utilized to take a closer look at the glass transition as an important fundamental feature of amorphous matter with relations to the processing and physical stability of small molecule drugs. With an additionally developed image and qualitative mode shape analysis, we are able to separate distinct features of the glass transition process and explain a previously observed two-fold change in resonance frequency. The results from this setup indicate the detection of initial relaxation to viscous flow onset as well as differences in mode responsivity and possible changes in the primary resonance mode of the string resonators. The modulated setup is helpful to distinguish these processes during the glass transition with varying responses in the frequency and quality factor domain and offers a more robust way to detect the glass transition compared to previously developed methods. Furthermore, practical and theoretical considerations are discussed when performing measurements on string resonators (and comparable emerging analytical techniques) for physicochemical characterization.

## 1. Introduction

Recent progress in the field of micro- and nanofabrication has paved the way for novel analytical tools to help investigate a variety of samples. Due to their high sensitivity, resonating microelectromechanical and nanoelectromechanical systems (MEMS, NEMS) of different geometries, such as strings, cantilevers, membranes, or trampolines, are specifically interesting in physicochemical characterization. 

Chien et al. have recently demonstrated this sensitivity advantage by employing a method that is able to probe single-molecule optical absorption by utilizing resonating nanodrums [1]. Furthermore, many techniques that aim to be employed in everyday chemical characterization are being developed. This includes measurements of the sample’s infrared absorption from resonating micromembranes or the probing of thermal characteristics of the sample using string and cantilever-based sensors [2,3,4,5,6,7,8,9,10].

We have shown the potential of resonating microstrings for the direct solid-state thermal characterization of a variety of samples with a method called Micromechanical Thermal Analysis (MTA) [9]. This instrumental technique is able to probe a small amount of material (typically in the pico- to nanogram range) to gain thermal and mechanical responses by tracking the resonance frequency and quality factor of the silicon-rich nitride (SiRN) strings during heating inside a vacuum chamber. Results from the initial setup have shown a wide applicability as well as further advantages in terms of sample size, sensitivity, and run times compared to conventional techniques such as differential scanning calorimetry (DSC). 

In this study, we introduce a modulated temperature version of MTA alongside an image and resonator mode shape analysis.

Common thermal techniques, which employ a temperature modulation such as modulated DSC (mDSC), are an important part in applied thermal analysis, for example in pharmaceutical development [11,12]. Furthermore, the working principle has recently also been applied in a miniaturized system on a deflection-based cantilever approach [13]. To the best of our knowledge, the principle has not yet been tested or applied on true resonance frequency-based measurements.

With the current study, we expand MTA in order to take an in-depth look at how the glass transition (Tg) manifests itself in MTA. The motivation firstly stems from the importance of the transition itself, for example for the processing and relation to the physical stability of amorphous drugs [14]. Furthermore, from a fundamental point of view, the amorphous state largely remains a mystery and several considerations surrounding the Tg have been established [15]. This includes the discussion of dynamic and static portions of the transition [16]. In this regard, an aim of the study was to further analyze a previously observed two-step change in resonance frequency during the glass transition process of small and large molecule samples [9]. More recently, the Tg has also been used as a go-to transition for investigation and validation in novel physicochemical characterization techniques based on nano- and microresonators [2,3,4,5]. Therefore, another aim of the study was to show what information can be gained and which challenges are to be considered in the routine physicochemical analysis of amorphous compounds and the Tg with MTA and comparable techniques. 

## 2. Materials and Methods

### 2.1. Materials and Sample Preparation

The well-known and often investigated low molecular weight organic model compound indomethacin [17,18], a pharmaceutical drug, was investigated in this study, and was obtained from Tokyo Chemical Industry Co., Ltd. (Tokyo, Japan). The experimental design comprised of recording multiple measurements with varying methodological conditions (e.g., sample size or different mode analysis, see also the results section below) of a single compound rather than studying multiple compounds. This was performed in order to probe the methodological space of the instrumental setup. As discussed later, intrinsic sample properties, such as the viscosity, influence the liquefaction process, and variations of such were therefore purposely excluded. An analysis and discussion of the applicability of MTA to various compounds is a central element of the aforementioned study, which employs the same sampling process [9]. 

Indomethacin was amorphized by standard quench-cooling: the drug was heated 10 °C above the melting temperature to 166 °C in an aluminum pan for three minutes and quickly cooled by transferring the pan onto a cold surface. The obtained glass was gently ground afterwards. 

### 2.2. Sensor Fabrication

**Figure 1:**
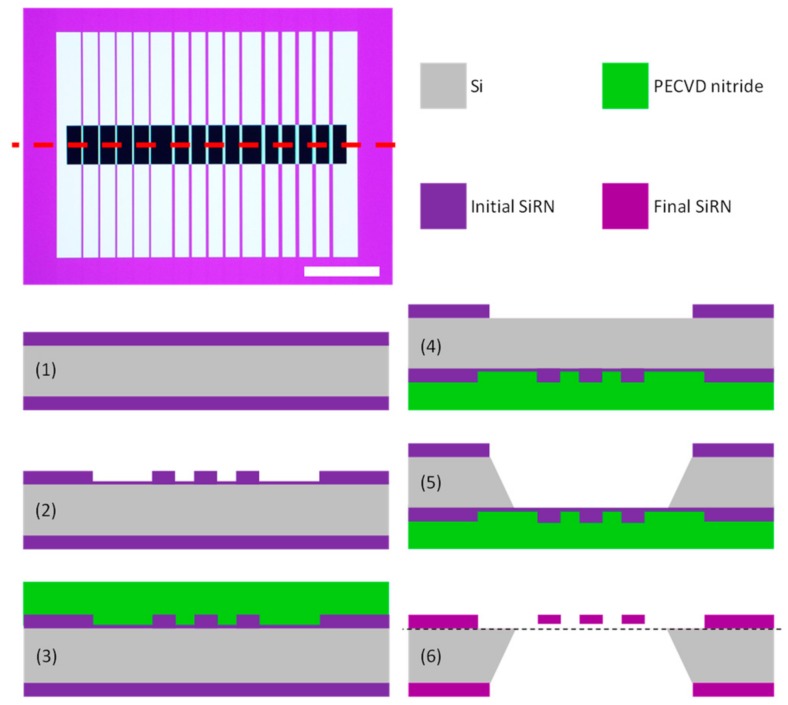
Process flow schematic. The cross-section of interest has been marked in the optical microscopy with a red dotted line. The image scale bar (bottom right) corresponds to 1 mm. (**1**) A silicon-rich nitride (SiRN) is deposited on a 350 µm thick double-side polished Si substrate by means of low-pressure chemical vapor deposition (LPCVD). (**2**) The SiRN on the front side is masked and slightly underetched, which leaves behind a residual nitride layer with a thickness of 5–10 nm. (**3**) A plasma-enhanced chemical vapor deposition (PECVD) nitride layer is deposited to protect the front side. (**4**) The SiRN on the back side is masked and the Advanced Oxide Etching (AOE) process goes all the way through the nitride. (**5**) An anisotropic KOH wet etch is conducted. (**6**) The PECVD nitride is stripped and the strings are finally released by buffered hydrogen fluoride (BHF) etching. Notice that the final SiRN thickness is slightly smaller than the initial thickness. The dashed line indicates the base level corresponding to the top surface of the Si substrate.

The micromechanical string resonators were produced on 4” double-side polished <100> Si substrates with a thickness of 350 µm ± 15 µm. The process flow is visualized in Figure 1 (see next page). Initially, a 193 nm thick silicon-rich nitride (SiRN) layer was deposited by means of low-pressure chemical vapor deposition (LPCVD, Tempress furnace). It is paramount that a low-stress nitride is deposited in order to maximize the yield of produced strings. Initially, the SiRN layer on the front side was masked and etched using conventional UV lithography (with flat alignment) and Advanced Oxide Etching (AOE, STS MESC Multiplex ICP). The substrates were pretreated with hexamethyldisilizane (HMDS) to increase resist adhesion and spin coated with 1.5 µm positive photoresist AZ^®^5214 E (MicroChemicals GmbH) prior to UV exposure (dose=110 mJ/cm^2^, Süss MicroTec MA6/BA6 aligner).

The resulting pattern was developed in a single puddle for 90 s before etching into the SiRN layer using C_4_F_8_ and H_2_ as the reactive gasses in the AOE. The etch was timed as to leave behind 5–10 nm nitride. This thin residual layer has two important ramifications that are outlined below. After the AOE etch, the resist was stripped using oxygen plasma ashing (300 Semi Auto Plasma Processor from TePla) and 7-up at 80 °C. The 7-up solution consists of concentrated H_2_SO_4_ with (NH_4_)_2_S_2_O_8_ added prior to substrate immersion. After the cleaning procedure, a 550–600 nm thick protective layer of silicon nitride was deposited on the front side by mixed frequency plasma-enhanced chemical vapor deposition (PECVD, SPTS Multiplex PECVD system). Using a mixed-frequency process has the advantage of reducing the intrinsic stress. After the PECVD process, the substrates were cleaned again using 7-up to eliminate particle contamination. Then, the SiRN on the backside of the substrates was masked and etched. The same UV exposure dose and development time were employed, and the AOE was used for etching all the way through (and slightly into the Si) the nitride layer. Once more, oxygen plasma ashing and 7-up were used for stripping the remaining resist, and the strings were pre-released by anisotropic KOH (28 wt%, <100> Si etch rate = 1.3 µm/min) etching at 80 °C. Due to the presence of the aforementioned thin nitride layer on the front side, the KOH etching time (4.5–5 h) becomes less crucial as the wet etch basically stops on the residual layer. Furthermore, the detrimental influence of pinholes in the PECVD protective layer is also eliminated as the KOH etch rate of LPCVD SiRN is exceedingly low. Pinholes serve as hydrophilic routes toward the underlying Si substrate, which resulted in a significant decrease in the resonator string yield in the absence of the residual skin layer. The final step in the process flow is the removal of the remaining PECVD nitride layer. This was done using buffered hydrofluoric acid (12 vol% HF with NH_4_F) at room temperature. The removal of the protective layer was monitored by eye and using optical microscopy (Nikon ECLIPSE L200), and the overall quality was ensured by scanning electron microscopy inspection (SEM, Zeiss Supra 40 VP). After this step, the strings are freely suspended and ready for measurements. Pictures of the finalized substrate and SEM images of the strings are provided in Figure S1 in the Supplementary Materials.

### 2.3. Instrumental Setup

The overall experimental setup, which consisted of a heating element inside a vacuum chamber, a thermocouple in contact with the microchip, and a piezoelectric element (NAC6024, Noliac A/S, Kvistgaard, Denmark) was adopted from [9]. The thermocouple output was recorded and stored with the help of a Raspberry Pi 2 microcontroller (Raspberry Pi Foundation, UK). The setup in this study was modified by using a Peltier element (Quick-Cool, Wuppertal, Germany) based heating, which allows the superimposing of a sinusoidal heating on the linear heating similar to mDSC [11]. In our method, the current and hence the temperature output of the Peltier element was controlled with a simple metal–oxide–semiconductor field-effect transistor (MOSFET). The gate of the transistor was addressed with pulse width modulation (PWM) generated by a microcontroller (Wemos D1 mini). The PWM duty cycle (DC) was created as follows:

(1) $$DC=k_{s}+k_{l}t+\sin\left(2\pi P^{-1}t\right)A_{\mathrm{mod}}$$

where *k_s_*: Peltier dependent minimum current constant, *k_l_*: linear heating constant, *t*: time, *P*: modulation period, and *A_mod_*: modulation amplitude

**Figure 2:**
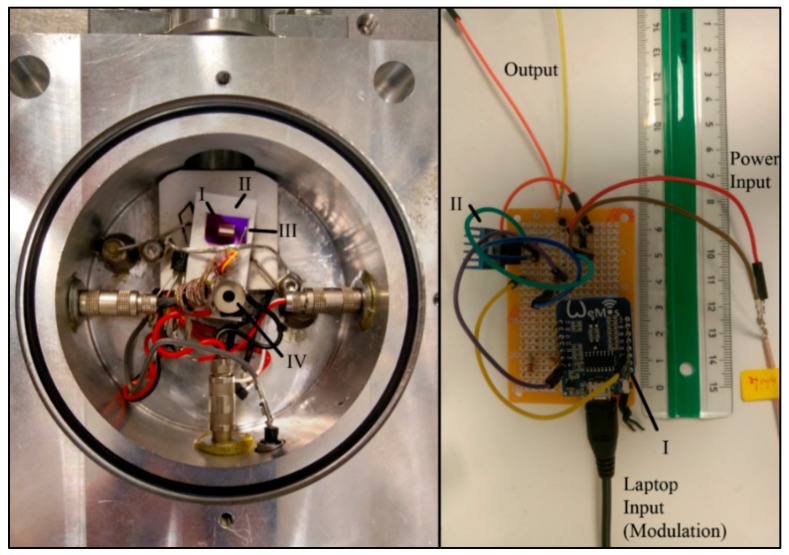
Left: Measurement chamber, **I**. Microchip, **II**: Peltier element, **III**: Thermocouple, **IV**: Piezo actuator, right: Controller used to create the temperature modulation, **I**: Microcontroller Wemos D1 mini, **II**: MOSFET.

Since the thermal inertia of the microsensors is very low, fast periods (20 s in this study) were used. *A_mod_* was set to achieve ± 0.2 °C and the underlying heating rate, defined by *k_l_*, was set to 1.6 °C/min. Figure 2 shows pictures of the instrumental setup. 

### 2.4. Data Analysis Process

The post-measurement data analysis was performed by first extracting the frequency data obtained from the read-out instrument, a laser-doppler vibrometer (MSA-500, Polytec GmbH, Germany) and the temperature data from the Raspberry Pi. The acquisition time of a single spectrum of the vibrometer was roughly 200 ms, which provides sufficient resolution given the low applied heating rate of 1.6 °C/min. A phase-locked loop in order to further increase time resolution was not used, as the possible drastic changes in the measured frequency and phase signal (see also the result section) permit a continuous phase detection in the feedback loop.

**Figure 3:**
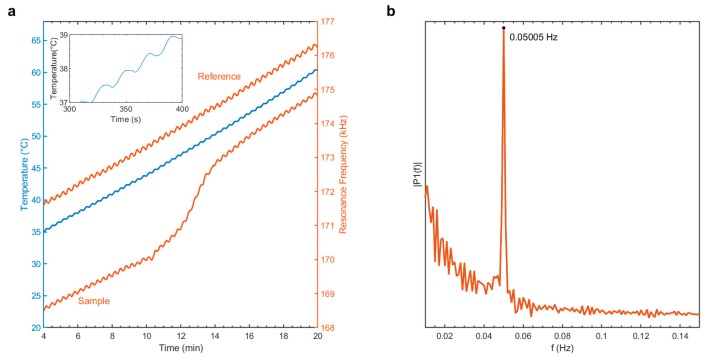
(**a**) Resonance frequency response of the reference (top) and sample (bottom) to the applied heating (middle). The inset shows a zoomed-in section of the heating profile, (**b**) Fast Fourier Transform of the measurement (sample signal of a) after detrend and normalization.

The applied heating profile (blue line) is shown in Figure 3a with a zoomed-in section shown in the figure inset. The resonance frequency response to the applied heating of an uncoated microstring (reference, top orange line) and the same string coated with indomethacin (sample, bottom orange) is also shown.

Fourier transforms of the frequency responses analyzed in this study (see Figure 3b for an example) show maxima at 0.050 Hz and therefore indicate a direct transfer of the applied period of 20 s. The resonance frequency follows the applied heating due to a temperature-induced change of tensile stress, which originates from the different thermal expansion coefficients of the SiRN string and Si microchip frame [9].

**Figure 4:**
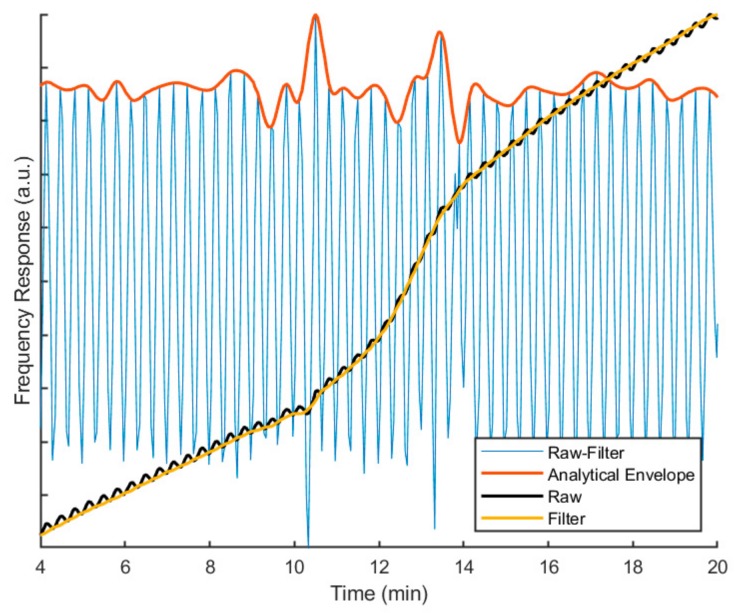
Overview of the applied deconvolution process. See manuscript description for more information.

The data deconvolution process, performed in MATLAB R2019a is presented in Figure 4. 

First, the raw frequency signal was averaged with a first-order Savitzky Golay filter (the filter framelength corresponds to the applied period). The filtered signal represents the none-periodic part and is called the underlying frequency response (UFS). The UFS was subtracted from the measured raw signal to obtain the periodic part. The upper envelope of the periodic part was extracted with the help of a modified Hilbert transform, which was—similar to practical data processing in conventional mDSC—obtained using a Fourier transform. Afterwards, the reversing signal (RS) was calculated from the upper envelope via the following equation:

(2) $$RS=\frac{P_{ue}}{TS_{ue}}\frac{P}{2\pi }$$

where *P_ue_*: upper envelope, periodic part, and *TS_ue_*: upper envelope of the measured temperature signal.

The RS contains information about signal responses that cause a deviation from the underlying modulation. These are further discussed in the results below. 

### 2.5. Image Analysis

**Figure 5:**
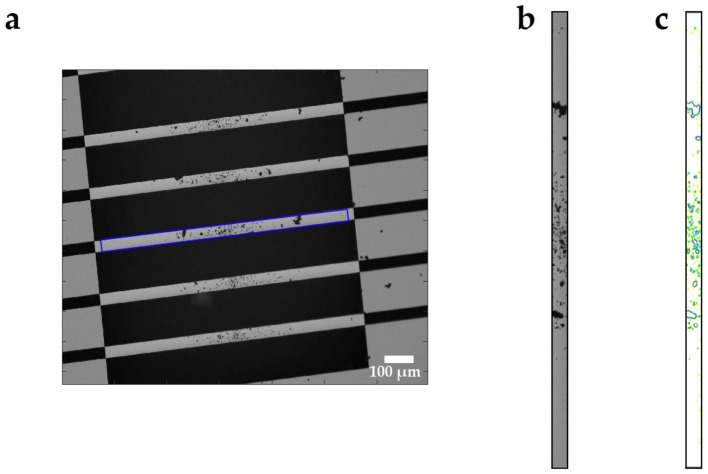
(**a**) Microscopic picture (from the vibrometer’s objective) of the lower part of a microchip showing five coated strings, each 50 µm in width. The blue frame highlights the string shown in (**b**) The contours of different levels of this string are shown in (**c**) Yellow represents the first level, green represents the second level, and purple represents the third level.

Moreover, image analysis was performed in MATLAB R2019a on the microscopic 8-bit grayscale pictures taken by the read-out instrument (laser-Doppler vibrometer from Polytec). Sample particles on top of the string resonators were detected by contour analysis on different levels, which allows a separation by particle size (see Figure 5). 

Typically, the first contour level detected particles with a diameter below 5 µm, the second detected particles between 5 and 10 µm, and the third detected particles above 10 µm. After level detection, the sample particle area inside the contours was registered by counting all the pixels, which are enclosed by the contours of the respective level. This was performed on all three levels and each image during the measurement and offers the possibility of tracking visual (two-dimensional) changes caused by shrinking or expanding the particles. The image analysis signals, which were obtained as pixel count for every level respectively, were filtered after the tracking. The image analysis is further visually explained in Chapter 1 of the supporting video, and an example of the applied filter is shown in the Supplementary Materials (Figure S2).

### 2.6. Grid Overlay Measurements

In order to visually assess the prominent resonance mode and changes thereof, a grid measurement of the coated string was recorded before and after each temperature ramp. The measurements were performed by first applying a grid consisting of 69 points, which were measured by the vibrometer, to the respective string on the chip. Post measurement, a point on the grid was selected to identify the resonance frequencies from the measured frequency spectrum. Afterwards, the vibrational amplitudes at every grid point were extracted for the given resonance frequency. An animation was subsequently made by superimposing a sinusoidal function to the amplitudes. Grid overlay measurements to visualize resonance mode shapes are further explained in Chapter 2 of the supporting video.

### 2.7. Notes on the Sampling Process and Further Methodological Details

The sampling procedure was adopted from [9]. For a more detailed explanation including a sampling chamber schematic, please refer to the reference. It should be noted that the employed sampling process is an inherently random solid-state deposition of the sample particles on top of the strings. After particle size reduction with a 30 µm sieve, a small vacuum was drawn inside the sampling chamber, which forces the sample particles to directly deposit on the string by inertial impaction. After the process, the strings were randomly covered with particles ranging from 2 to 25 µm, which were predominantly located between ¼ and ¾ of the length of the string. Sample sizes can roughly be controlled by adjusting the vacuum drawing time and range from 0.3 to 10 ng. 

Measurements in this study were performed at 1–2 mbar. The medium vacuum range was selected to maintain the practical character of the method, as a higher vacuum can lead to a decomposition of the small molecule samples.

The reader is referred to reference [9] for further methodological details, including the calculation of the quality factor. The following equation—where *f_0_* is the initial resonance frequency of the uncoated string, *f* the resonance frequency of the string covered with sample, and *m_0_* the mass of the SiRN string—was used to approximate the sample mass (for a derivation, please refer to the supporting information of [9]):

(3) $$m_{sample}=\frac{\pi m_{0}\left(f_{0}^{2}-f_{res}^{2}\right)}{\left(\pi +2\right)f_{res}^{2}}$$

### 2.8. Modulated DSC (mDSC) and Dynamic Mechanical Analysis (DMA)

Modulated DSC measurements were performed on a Discovery DSC (TA Instruments, New Castle, DE, USA) under a nitrogen gas flow of 50 mL/min. Samples were prepared as described in Section 2.1, and 3 mg were crimped into aluminium sample pans. The samples were heated at a rate of 1.6 °C/min with a modulation amplitude of 1 °C and a period of 60 s. Reported Tg values (midpoint) were determined with TRIOS software (version 4.1). 

DMA measurements were performed on a Q800 DMA (TA Instruments, New Castle, DE, USA) with a dual cantilever clamp and a powder holder setup. Samples were prepared as described in Section 2.1. The measurements were recorded with a frequency of 0.1 Hz, a deformation amplitude of 20 µm, and a heating rate of 1.6 °C/min.

## 3. Results

### 3.1. Glass Transition Detection and Signal Comparison

**Figure 6:**
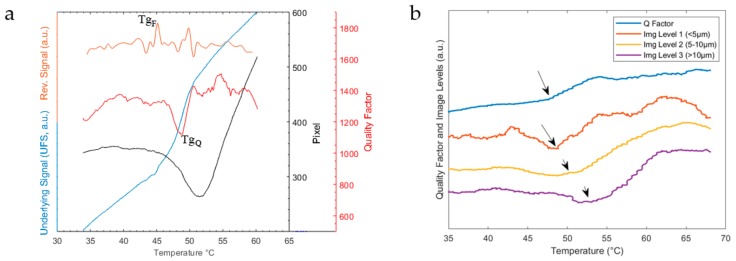
(**a**) Micromechanical Thermal Analysis (MTA) responses of the underlying frequency signal (light blue), the reversing signal (orange), and quality factor (red) as well as the response from the 2D image analysis of level 2 as pixel count (black) during the glass transition process. Tg_F_ marks the Tg as detected by the local maximum in the reversing signal, and Tg_Q_ marks the Tg as detected by the quality factor minimum. (**b**) Image contour levels obtained from the image analysis and the quality factor from another measurement example. The main slope onsets during the glass transition are marked with an arrow, respectively.

The employed methods lead to multiple signals originating from a single measurement. A comparison of the UFS (blue), the RS (orange), the quality factor (red), and the image analysis signal (black, level 2) of a single measurement is given in Figure 6a.

The onset of the glass transition process is detected by a local maximum at 45 °C (marked with Tg_F_) in the RS. This temperature coincides with the temperature obtained from the initial slope change in the frequency domain (here UFS), which was previously used to determine the onsets of thermal phase transitions [3,9]. A second maximum is present at a slightly higher temperature of 50 °C. While the initial maximum is consistently present in all measurements in this study, the presence of further maxima is dependent on the detection of multiple changes during the Tg (see also the paragraph below and discussion section). The present example shows a considerable detuning in the UFS caused by the effective mass changes of the micro resonator [9], which in turn are caused by the viscous flow of the sample. While the overall mass stays constant, the effective mass is dependent on the shape of vibration. In agreement with these observations, a drop in quality factor (Q) is detected, followed by a sharp increase, marked with Tg_Q_ in Figure 6a. The Q corresponds to the viscoelastic material damping [9,19]. During the glass transition, the sample viscosity drops, which enables viscous flow on top of the string resonator [20]. This is further underlined by the response of the image analysis, which—after an initial shrinkage of the particles—detects a steep increase in the sample particle pixel area. The viscous flow of the sample is detected as a widening (due to liquefaction) of the particles via the 2D top view image analysis. While the quality factor and the image analysis signal show comparable responses, their minima, when compared, are slightly different in temperature. 

This is due to the time, which is needed to heat up the particles until a pure widening is detected via image analysis. Figure 6b compares the quality factor to the image analysis on all three contour levels for another measurement example. The lower the contour level and therefore particle size, the closer the minima of the image analysis to the instant response of the quality factor. This example also shows that the quality factor does not necessarily undergo a very pronounced minimum but always shows a shift toward higher values at the Tg.

The glass transition as detected by Tg_Q_ is consistently recorded at higher temperatures than the Tg_F_. Across all the measurements of this study, which did not include a change in prominent resonance shape, the difference was + 5.0 °C (± 1.3 °C, n = 12). 

### 3.2. Comparison with Conventional Techniques

**Figure 7:**
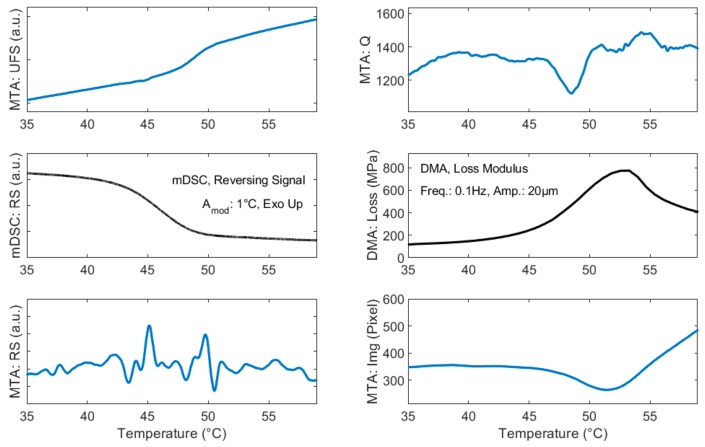
Comparison between MTA signals (in blue) and conventional techniques (in black). All x-axes are linked and represent temperature (°C) as indicated at the bottom of the figure. All measurements were performed with a heating rate of 1.6 °C/min.

A comparison of the MTA signals with conventional techniques, namely mDSC and DMA, is given in Figure 7. The glass transition as measured by TgF (43.93 ± 1.11 °C, n = 12) corresponds well to the Tg as measured by mDSC (44.87 ± 0.66 °C, n = 3) with the same heating rate.

Similarities in the response of Q and the signal obtained from image analysis with DMA, a more dynamic measure of the Tg, can further be seen in the right column of the figure. It should be noted that a comparison of absolute values between Tg_Q_ or image analysis with DMA is aimless, as DMA experiments rely both on the used heating rate and the input frequency. As a kinetic transition, the glass transition shifts to higher values with higher applied frequency [21]. Since MTA and DMA operate at different frequencies, an absolute comparison is therefore limited. 

The temperature range obtained for the Tg of indomethacin both from MTA and the conventional techniques in this study matches well with the Tg values reported in the literature [17,18].

Similarities between MTA signals and conventional techniques are further analyzed in the discussion section below. 

### 3.3. Variance through Sample Size and Sample Position

**Figure 8:**
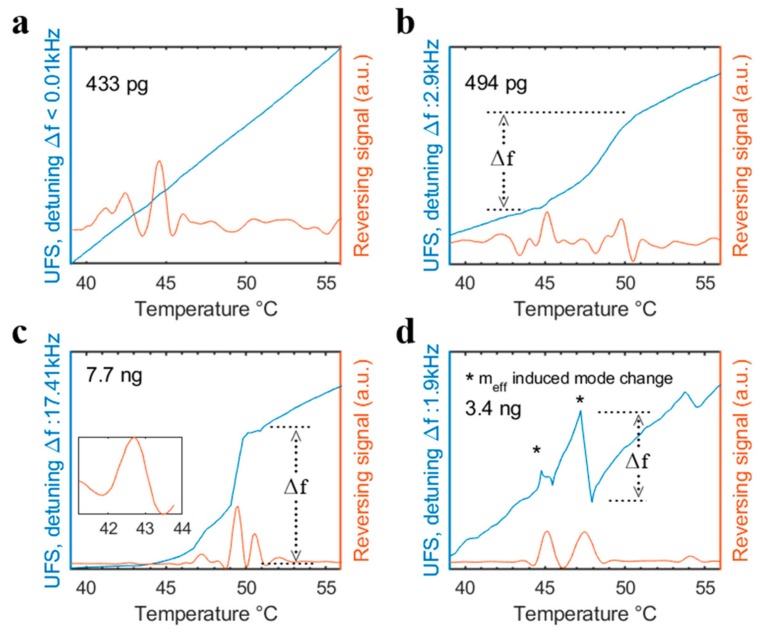
Comparison of modulated MTA signals during the Tg at low (**a** and **b**) and large (**c** and **d**) sample sizes. Δf marks the maximum detuning during the Tg. The inset in c shows a zoomed-in section of the reversing signal at the Tg onset.

Figure 8a,b shows measurements on the lower end of the sample size (433 pg and 494 pg, respectively) spectrum for MTA. 

In contrast to the response of Figure 8b (as described above in Figure 6a), the example of Figure 8a does not show a pronounced detuning in the underlying frequency signal. Only a very slight slope change in the UFS is recorded. However, the modulated setup is able to detect a local maximum at around 45 °C indicating the Tg_F_. This highlights the potential of the modulated setup to further push the sample size limits of MTA and increase the responsivity for specific thermal events such as the Tg. Even though similar sample sizes are used and the tracked frequencies originate from a similar resonance mode shape, the responses of Figure 8a,b are very different. As noted previously [9], this can be attributed to the effective mass changes of the resonator. The sampling technique randomly covers the two mentioned string resonators of measurement Figure 8a,b with the sample. For a single given resonance mode, the different location of the sample particles can significantly influence the system. For example, a big particle close to a nodal point of the given mode exhibits a different influence on the effective mass and hence frequency responsivity compared to multiple particles with the same absolute mass spread out over the string. This holds especially true for thermal events that include liquefaction of the particles such as the glass transition process.

### 3.4. Variance of Different Resonant Modes

**Figure 9:**
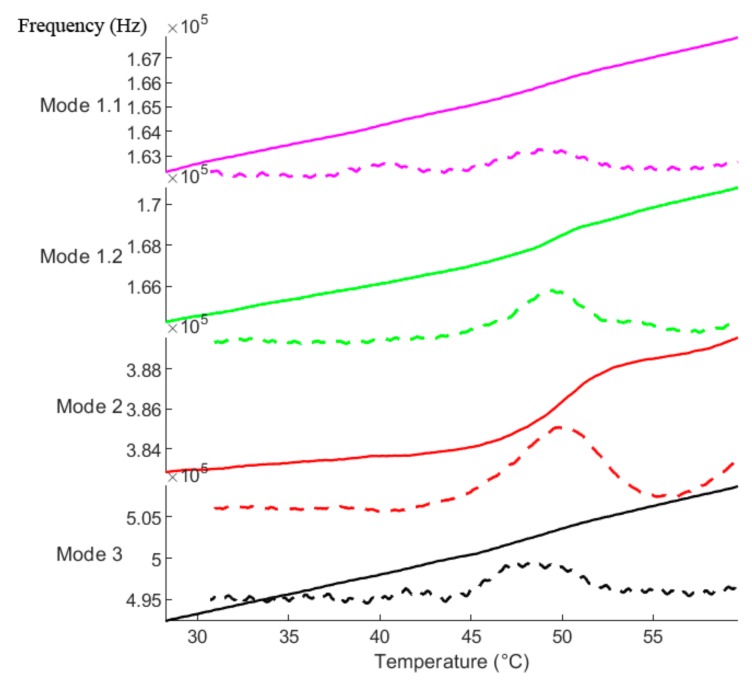
Four different modes of a single string during the glass transition process. The dashed lines represent the respective first derivative of the tracked modes.

The same principle also applies vice versa, when investigating multiple modes of *one single string* covered with a sample. While the sample mass is located at the same position for all modes, the effective sample mass (which takes the resonance shape with different nodal points into account) differs heavily. A measurement example of four modes from a single string, continuously tracked during the Tg, is given in Figure 9.

Mode 1.1 and mode 3 represent the first and third harmonic flexural mode (see also Chapter 2 of the supporting video) of the string, respectively. Hence, the resonance frequency of mode 3 (493 kHz) at the start of the measurement is approximately equal to three times the resonance frequency of mode 1.1 (163 kHz). Mode 1.2 and mode 2 are “mixed” modes (see the Discussion section for terminology) of the first and second harmonic respectively, each with significant torsional involvement. For clarification, we have supplied another figure in the Supplementary Materials (Figure S3) including a scaled color image plot of this measurement, which further shows the frequency domain as an overview with and without the tracked modes. 

As seen in Figure 9, each of the above-mentioned modes shows a different responsivity toward the glass transition process. To highlight the differences in the frequency detuning, the first derivatives of the respective modes are also given in the figure as dashed lines. While all modes show a response at the glass transition temperature, the detuning of mode 1.2 and 2 is considerably higher than the detuning of mode 1.1 and 3. In general, the local mass responsivity scales with the square of the local vibrational amplitude of a given resonance mode [9]. Since the sampling technique randomly covers the strings with particles of different sizes and shapes, a quantitative description of the exact frequency changes is not possible. This highlights the complexity in analyzing the glass transition and the need to identify different modes of a single string alongside the development of optimized sampling procedures to better describe the observed frequency changes. 

Measurement examples on the upper end of the sample size range are shown in Figure 8c,d. Figure 8c shows a large detuning in the UFS response and several responses in the RS. While Figure 8c was continuously tracked and a change in resonance mode shape was not recorded, Figure 8d shows switches in the prominent mode, which are marked with an asterisk in the figure. Here, the prominent mode is simply defined as the mode with highest magnitude in a given frequency range. Despite showing that more sample mass subsequently leads to a stronger frequency detuning on a given mode, these results also highlight that large sample masses can but must not necessarily induce a change of the prominent mode. Since the visualization of three-dimensional modes and their respective changes is limited in a two-dimensional printed paper, the authors have also added an additional animation of a grid measurement in Chapter 3 of the supporting video. In this measurement, a lower resolution grid consisting of 21 points was applied to a heavily crowded string. The grid was consecutively measured at every frame during the measurement. The animation shows, that, especially for measurements with large sample quantities, the prominent mode can change several times during a single measurement of the Tg.

## 4. Discussion

The difference between Tg_F_ and Tg_Q_ as described in Section 3.1 is consistent with previous micromechanical studies [3,9] and is often explained by two fundamental features of the glass transition process, which are named the static (or thermal) and dynamic glass transition [16]. The former typically relates to thermodynamic quantities such as the heat capacity, volume, or density and is measured by techniques such as DSC [11,21] or dilatometry [21], while the latter is more kinetic in nature and measured by techniques such as dynamic mechanical analysis [21,22], dielectric spectroscopy [21,23], or light/neutron scattering [24]. The assumption that modulated MTA is able to probe both these characteristics of the glass transition is further underlined by the comparison to the conventional techniques in Section 3.2, as well as from the fact that the image analysis signal approaches the instant response of Tg_Q_ for small particles (see Figure 6b). 

Hence, we argue that the initial signal from the reversing signal of the modulated setup, namely Tg_F_, corresponds to the initial (static) change during the glass transition, whereas Tg_Q_ corresponds to the liquefaction process, which can be seen as a result of the primary change in the intermolecular landscape. 

Our study further confirms this dual nature of the glass transition; nonetheless, the glass transition is herein not denoted as static or dynamic, as this phonetically might imply a fundamental separation of these processes. However, their exact interplay is an open question surrounding the glass transition [15,16].

As seen in the Results section, the reversing signal can (but most not necessarily) contain more information than the initial change during the glass transition. While the signal is always sensitive to the initial change, subsequent maxima do not always simply correspond to the end of the transition (see also Figure 8). The modulated setup is rather responsive both to the initial change during Tg but also to subsequent changes in the prominent resonance mode (see Figure 8d) or the initial change caused by effective mass variations (see Figure 8c), which cause a strong detuning. These observations further qualitatively explain the origin of the two-fold change in resonance frequency observed in the previous study [9]. The modulated setup offers a more robust way to visualize these changes during the glass transition, as well as a stronger responsivity to the initial change (see Figure 8a) than the pure change in resonance frequency slope, which was used previously to describe thermal transitions.

The observed mode shapes as well as changes thereof during the glass transition further raise interesting questions. As mentioned in Section 3.4, the initial mode shapes, which show a stronger responsivity, are termed “mixed” modes. This implies that these modes deviate from pure flexural (or pure torsional) modes in a plain visual observation of the animations obtained from grid overlay measurements (see also the last chapter of the supporting video). These modes were shortly observed in the previous study [9] and termed “coupled” modes. However, the exact origin of these modes remains an open question. Intermodal coupling normally occurs in the presence of strong non-linearity, which can be caused by intrinsic (e.g., material, geometry, actuation, detection) properties of the system or be purposely introduced to exploit the non-linear regime [25,26]. On the other hand, the observed mode shapes in this study might only be “local normal modes”, which originate from a non-uniaxial stress field of the relatively wide strings used in this study. Therefore, further studies that employ more narrow strings might not show such mode shapes (in similar frequency regions as the normal modes). However, while being a study on its own, this approach also hinders the practicality of the method, as the wide strings are purposely chosen to facilitate the easy sampling procedure. 

Our study further highlights the complexity in the analysis of thermal transitions in MTA with a random solid-state sampling procedure. Both from a practical and theoretical standpoint, the following factors should be considered:

- The mode shape of resonance, as different modes exhibit different responsivities toward the glass transition process
- The fact that the prominent resonance mode might change during processes such as the glass transition
- The sample particle distribution on the string, as it determines the effective mass
- The temperature offsets of quality factor and frequency domain, representing the dual nature of the glass transition
- The applied sample mass and particle diameter, as a higher sample mass or diameter increases the probability of significant effective mass changes during thermal events that include liquefaction.

The above listed considerations further underline the importance of sampling techniques in novel nano- and micromechanical sensor-based applications for physicochemical analysis. As described, the random sample particle deposition can lead to a multitude of signals and while changes in the sample can be explained qualitatively, the sampling complicates a quantification of the underlying processes.

The applied sampling technique has the advantage of being a fast and easily applicable flow-through process, which can be implemented in high-throughput devices to complement the other advantages of MTA. Furthermore, the sampling technique does not physically alter the sample, which is a major benefit compared to other available sampling techniques for MTA such as spray or spin coating, ink jet printing, or melt infiltration [2,3,4]. Nonetheless, further development of MTA is in need of an inert high-precision sampling technique or geometry optimization in order to facilitate the step toward quantification without losing the benefits of the previously developed MTA variants.

## 5. Conclusions

In this study, we have applied a temperature modulation program to resonance frequency-based measurements of microstring resonators, as well as an image and mode shape analysis with successful integration in the data analysis process. This enables the sensitive detection of the glass transition of organic amorphous compounds and is further helpful to distinguish different processes during the glass transition with varying responses in the frequency and quality factor domain. Furthermore, the modulated setup is able to offer a more robust way to visualize the responsivity toward different parts of the glass transition process.

Our study also discusses and highlights a general need in emerging miniaturized analytical devices: an emphasis has to be set on the sampling procedures, as they have to keep up with the miniaturization of the technique itself. As more variants of MTA (and comparable techniques) are being developed, a proper description of the resulting sample morphology and location on the resonating geometry is needed to analyze similarities and differences in the respective results.

## Supplementary Materials

The following are available online at https://www.mdpi.com/1424-8220/20/4/1019/s1, Figure S1: Substrate and SEM images of the fabricated microstring sensors, Figure S2: Image analysis filter example, Figure S3: Different responsivity of various modes including frequency domain overview.
