# Supplementary material for: Temperature-Modulated Micromechanical Thermal Analysis with Microstring Resonators Detects Multiple Coherent Features of Small Molecule Glass Transition
Source: Sensors (Basel). 2020 Feb 13;20(4):1019. doi: 10.3390/s20041019 (PMC7070930; doi:10.3390/s20041019)
Supplement: Supplementary file 1 [file sensors-20-01019-s001.pdf]

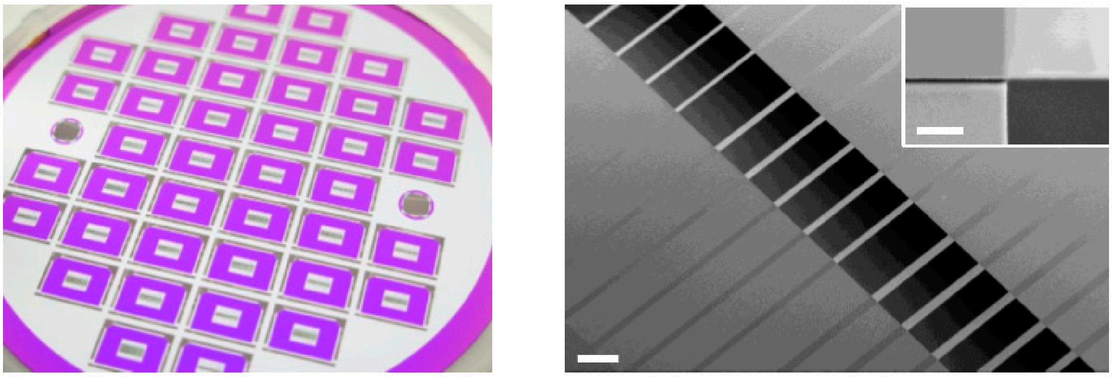

Figure S1. Camera picture and scanning electron microscopy (SEM) images of the produced micromechanical string resonators. Left: picture of a substrate after the final PECVD nitride etch, showing the 40 individual chips. Right: SEM image, acquired at a tilt angle of 30°, showing an overview of several strings. The scale bar corresponds to 200 µm. The inset, acquired at a tilt angle of 46°, shows a zoom-in on the anchor point where the thin strings are attached to the Si substrate. The inset scale bar corresponds to 2 µm.

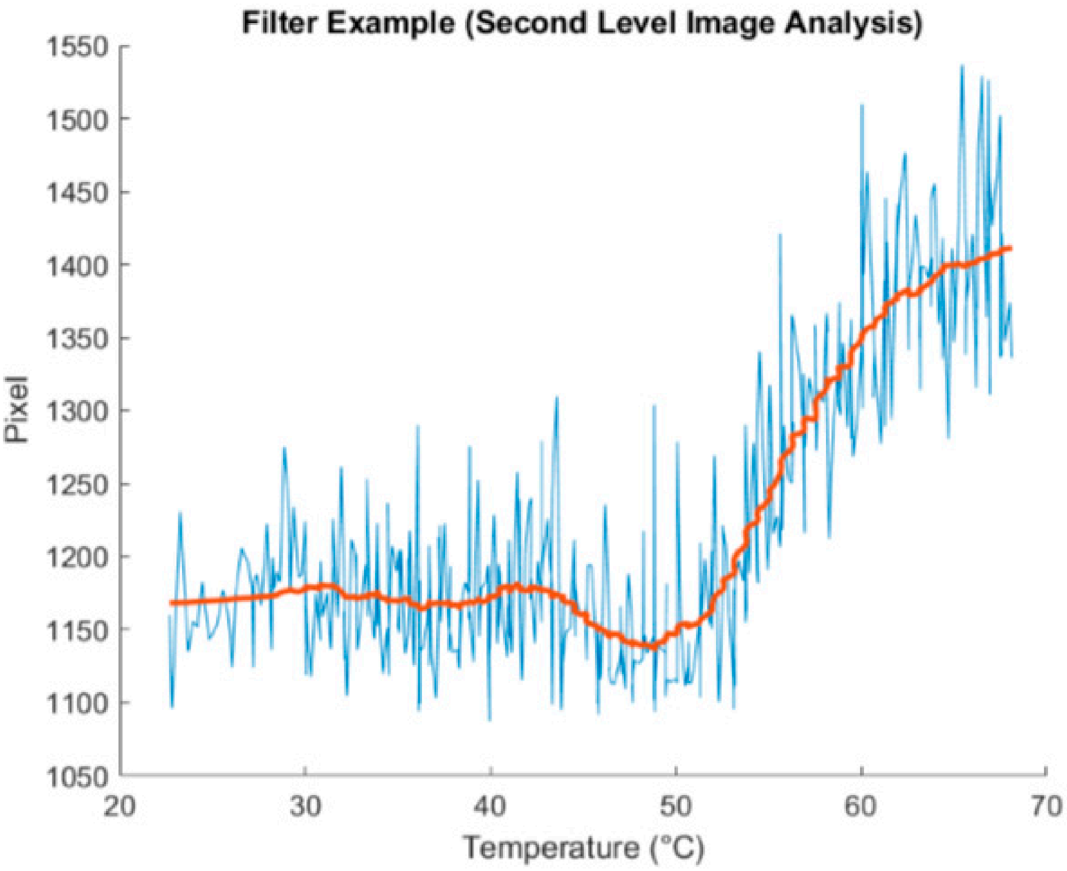

Figure S2. Measurement example of the applied filter (orange) to the raw pixel count signal (blue) obtained from the image analysis.

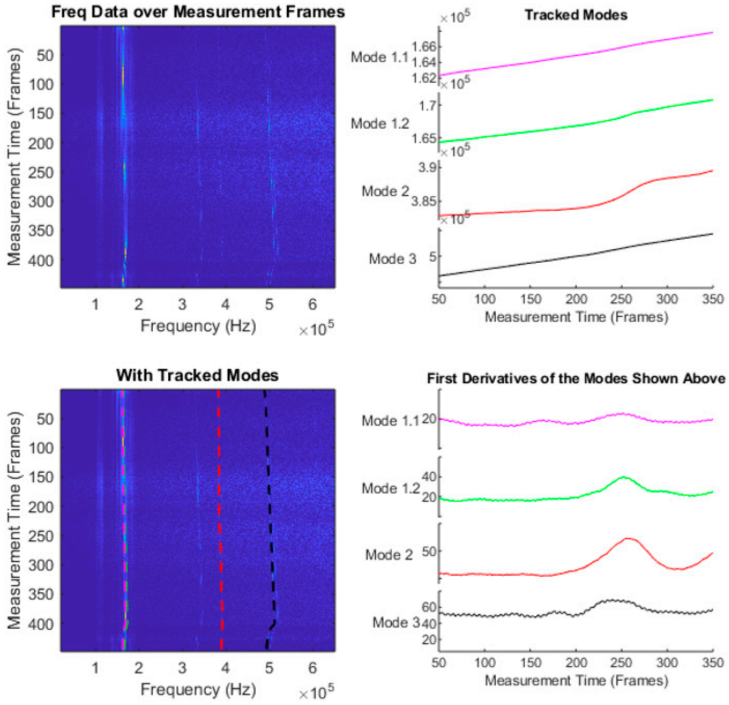

Figure S3. Left, top: Overview of the raw frequency data over the measurement frames of a single measurement. Left, bottom: Same overview with tracked mode examples. Right, top: Stacked plot of the different tracked modes. Right, bottom: First derivative of the tracked plots to highlight the different response to the slope changes during the glass transition at around frame 250 (equal to 49°C).
